# The impacts of ocean acidification, warming and their interactive effects on coral prokaryotic symbionts

**DOI:** 10.1186/s40793-023-00505-w

**Published:** 2023-06-07

**Authors:** Jinlong Li, Guangjun Chai, Yilin Xiao, Zhiyong Li

**Affiliations:** State Key Laboratory of Microbial Metabolism, School of Life Sciences and Biotechnology, Shanghai Jiao Tong University, Shanghai, 200240 People’s Republic of China

**Keywords:** Coral prokaryotic symbionts, Metatranscriptome, Differentially expressed genes, Interactive effect, Acidification, Warming

## Abstract

**Background:**

Reef-building corals, the foundation of tropical coral reefs, are vulnerable to climate change e.g. ocean acidification and elevated seawater temperature. Coral microbiome plays a key role in host acclimatization and maintenance of the coral holobiont’s homeostasis under different environmental conditions, however, the response patterns of coral prokaryotic symbionts to ocean acidification and/or warming are rarely known at the metatranscriptional level, particularly the knowledge of interactive and persistent effects is limited. Using branching *Acropora valida* and massive *Galaxea fascicularis* as models in a lab system simulating extreme ocean acidification (pH 7.7) and/or warming (32 °C) in the future, we investigated the changes of in situ active prokaryotic symbionts community and gene expression of corals under/after (6/9 d) acidification (A), warming (H) and acidification–warming (AH) by metatranscriptome analysis with pH8.1, 26 °C as the control.

**Results:**

A, H and AH increased the relative abundance of in situ active pathogenic bacteria. Differentially expressed genes (DEGs) involved in virulence, stress resistance, and heat shock proteins were up-regulated. Many DEGs involved in photosynthesis, carbon dioxide fixation, amino acids, cofactors and vitamins, auxin synthesis were down-regulated. A broad array of new DEGs involved in carbohydrate metabolism and energy production emerged after the stress treatment. Different response patterns of prokaryotic symbionts of massive *G. fascicularis* and branching *A. valida* were suggested, as well as the interactive effects of combined AH and persistent effects.

**Conclusions:**

The metatranscriptome-based study indicates that acidification and/or warming might change coral’s in situ active prokaryotic microbial diversity and functional gene expression towards more pathogenic and destabilized coral-microbes symbioses, particularly combined acidification and warming show interactive effects. These findings will aid in comprehension of the coral holobiont’s ability for acclimatization under future climate change.

**Supplementary Information:**

The online version contains supplementary material available at 10.1186/s40793-023-00505-w.

## Background

Coral reefs ecosystem, one of the highly diverse and valuable ecosystems on earth, is under increasing threat from the global climate change caused by CO_2_ emission. For instance, the annual sea surface temperature increased at a rate of 0.038–0.074 °C/year in recent decade, and pH decreased at a rate of 0.012–0.014/year in two coastal waters of the South China Sea [1]. According to the prediction of Intergovernmental Panel on Climate Change (IPCC), ocean pH will decrease to ~ 7.8–7.7 at the end of the 21st century [2], the global temperatures will rise at least 2 °C during 2050–2100 [3]. Ocean acidification and warming from increasing levels of atmospheric CO_2_ represent the major threats to coral reefs, causing widespread coral bleaching, reducing the rates of calcification for corals [3], and inevitably leading to loss of reef habitat and species biodiversity.

In the coral reef ecosystem, healthy corals are fundamental ecosystem engineers, constructing the productive and sustainable reef ecosystem which support ~ 25% of all described marine species [4]. The corals, with diverse symbiotic microbes and protists including Symbiodiniaceae, are collectively termed the coral holobionts [5]. These prokaryotes can provide the holobiont with essential nutrients such as carbon [6], nitrogen [7], sulphur [8], phosphate [9], vitamins [10], and metals [11] to their coral host. In addition, these prokaryotes can contribute to the host immunity via the production of antimicrobials, preventing invasion of pathogen [12]. Thus, coral microbiome strongly influences coral health and survival. Meanwhile, coral microbiome plays a key role in the acclimatization and maintenance of the coral holobiont’s homeostasis under different environmental conditions, and importantly, may contribute to coral holobiont’s resilience to environmental stress [13, 14].

In the face of global climate change such as ocean acidification and warming, it is important to understand the mechanisms contributing to coral holobionts resilience. Beyond coral physiological acclimatization, coral microbial symbionts can respond to changing environmental conditions. Earlier studies of coral microbiology proposed a coral probiotic hypothesis [15], wherein a dynamic relationship exists between corals and their symbiotic microorganisms, selecting for the coral holobionts that is best suited for the prevailing environmental conditions [15]. The coral holobionts can adapt to the changing environmental conditions more rapidly and with greater versatility than a process that is dependent on genetic mutation and selection of the coral hosts by altering the structure of coral-resident microbial community or the metabolic capabilities [15–18]. Pootakham et al. [19] investigated the dynamic bacterial and algal communities throughout a natural bleaching event, and provided evidence of significant changes in the structure and diversity of *Porites lutea*-associated microbiome during thermal stress. McDevitt-Irwin et al. [20] evaluated the impacts of the top three stressors facing coral reefs (climate change, water pollution and overfishing) on coral microbiome, and suggested that microbial community plays important roles in the ecological resilience of corals, and encouraged a focus on the microbial contributions to resilience for future research. van Oppen et al. [21] synthesized the current understanding of coral-associated microbial community, the drivers shaping the microbial diversity, and the role of the microbiome in acclimatization and adaptation of the host to climate warming. It is accepted that coral microbiome can be harnessed to assist the future persistence of coral reefs and provide novel perspectives for the development of microbiome engineering and its implications for coral reefs conservation and restoration. However, compared with the knowledge of Symbiodiniaceae community align with differences in stress sensitivity in corals, the role of microbes in coral thermal/acidification resilience is rarely known [22, 23], the microbial contribution to ecological resilience is still largely overlooked in coral reef ecology. The exact mechanisms by which the coral microbiome supports coral health and increases resilience are poorly understood. Meanwhile, ocean acidification and warming occur simultaneously rather than by oneself. However, most researches on the coral microbiome’s response always focus on one stress [17, 22, 23], studies on the combined effects of acidification–warming on coral holobionts are still rare [24–26], very few reports tackle the functional or metabolic changes of these assemblages based on gene expression [26]. In particular, it largely remains unknown whether the microbial community’s structure and function can recover after espousing to these stresses. Therefore, it is very essential and important to reveal the molecular response of coral microbiome to combined ocean acidification and/or warming, the following questions need to be addressed: (1) how the coral microbial community structure and function response to acidification and/or warming? (2) whether the response of coral microbiome is coral species dependent? (3) whether the coral microbiome could recover after the stress removal? (4) are there any superimposed or palliative effects of combined acidification-warming?

Massive *G. fascicularis* is the dominant coral species in the South China Sea, and was reported as stress-tolerant coral [26]. Branching *A. valida* is a stress-sensitive coral species, that becomes rare from previously dominate species in the South China Sea because of human activity and environmental change [27]. For a better understanding of how coral microbiomes respond to the extreme ocean acidification and/or warming in future, using branching *A. valida* and massive *G. fascicularis* as models in a lab simulation system, we examined the in situ active (i.e. with transcriptional activity) prokaryotic symbionts diversity and functional gene expression profiles under/after (6/9 d) single or combined acidification and warming by meta-transcriptomics analysis, particularly assessed the synergistical effects of combined AH. The findings from this study highlight the prokaryotic diversity and functional variation of various coral species under the single or combined environmental stress which will aid in comprehension of the coral’s ability for acclimatization under future climate change.

## Materials and methods

### Sample collection and short-term stress experiment

The corals used in this study were collected from Xuwen National Coral Reef Nature Reserve in the South China Sea (N 20° 15′ 29″ E 109° 54′ 28″) in October 2013 with the permission of local government. Approximately 20 small colonies of each coral were collected at ~ 8 m depth from an offshore reef (~ 1 km offshore) by Scuba diving. Considering the genetic and the environmental variance, all colonies were collected from the same reef. After collection, corals were cut into 5 cm^3^ fragments. The fragments were immediately transported to the aquaculture facilities at the local reserve monitoring station in Xuwen, where they were maintained in a flow-through seawater system (seawater temperature: 26 °C, salinity: 3.3%, pH: 8.1, flow: ~ 30 L/h) and allowed to acclimate for 7 days. After acclimation, only the apparently healthy coral specimens were chosen for the next short-term heat/acidification stress experiments.

**Fig. 1:**
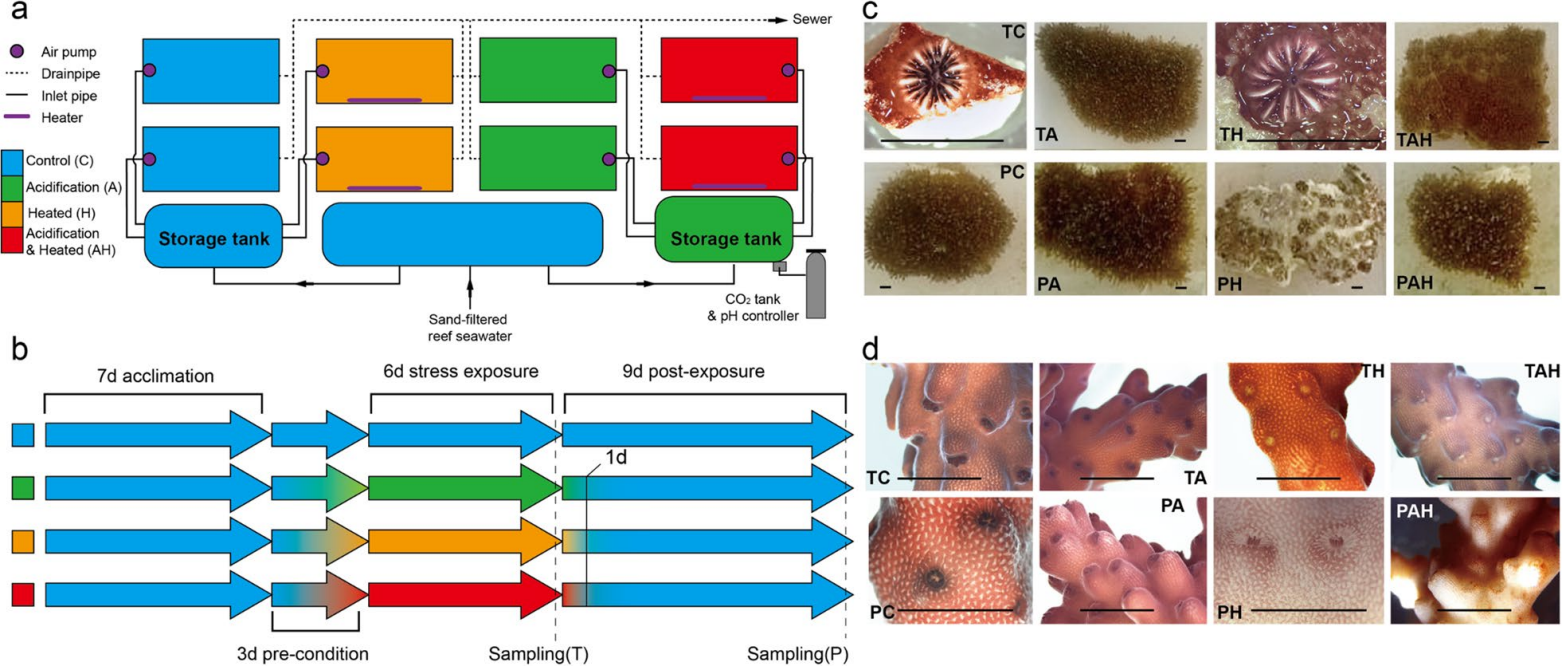
Experimental system and morphological observation. **a** System design; **b** Stress treatment and sampling process; **c** Morphological response of *G. fascicularis* under/after stress; **d** Morphological response of *A. valida* under/after stress. The rulers represent 1 cm scale. T: under stress; P: after stress; A: acidification; H: warming; AH: acidification–warming

Replicate fragments (n = 10) were randomly assigned to one of two experimental tanks (~ 100 L) per condition (i.e. C: control; A: acidification; H: warming; AH: acidification–warming) per coral. The simulation system consisted of 16 flow-through tanks (~ 100 L) receiving sand-filtered reef seawater (Fig. 1a). Acidification was manipulated by bubbling pure CO_2_ and controlled by using a pH controller (UP-aqua, Taiwan). When reservoir pH level exceeded the target value for the acidification treatment, the controller opened a valve that delivered CO_2_ gas until target value was restored; Temperature level was regulated by aquarium heaters (EHEIM, German). Submerged-pumps maintained water movement, and all tanks were illuminated under a 12:12 h diurnal cycle at 180–200 mmol photons m^− 2^ s^− 1^ (55 W, 10,000 K compact fluorescent tubes). To minimize stress attributable to sudden environmental changes and reduce physiological shock, 3days pre-condition treatment was performed to bring corals to the experimental or the control levels gradually.

The experimental CO_2_ level matched the category VI for IPCC CO_2_ stabilisation scenarios, with peaking CO_2_ in years 2060–2090 [2]. In general, coral will bleach when seawater temperature rises to levels above 30 °C [28], thus the experimental temperature is designed as 32 °C in order to evaluate the impact of extreme thermal stress in the future. The tanks in control treatment were maintained at ambient temperature and pH condition (temperature: ~ 26℃, pH: ~ 8.1). The summary of parameter values used in the experiment was in Additional file 1: Table S1. The response to stress in a natural coral community was not measured because the seawater pH 7.7 and temperature 32 °C are predicted by the Intergovernmental Panel on Climate Change (IPCC) at the end of the 21st century [2], no coral samples under these stress condition can be found in natural oceans.

Sampling was conducted after 6 days of exposure to the stress conditions (Fig. 1b). The 6 days (hereinafter to be referred as T) time point was chosen because obvious morphological changes (coral fragments paled or bleached) were observed in the H group *from G. fascicularis* (Fig. 1c) and in the H and AH groups from *A. valida* (Fig. 1d). To assess the recovery potential of corals, we conducted a post-treatment experiment in which all the stressors were removed. The second sampling was taken after 9 days of post-treatment. The 9 days (hereinafter to be referred as P) time point was chosen based on that no sign of recovery was observed. The coral samples at T and P time were selected and incubated in 10-fold volume RNAlater (DongSheng, China) overnight at 4 °C, and stored at − 80 °C.

### RNA extraction, purification, quantitation and cDNA synthesis

For RNA isolation, coral fragments were ground in liquid nitrogen using a mortar and pestle. Total RNA was extracted from ~ 500 mg of the homogenized sample using RNeasy Plant Mini Kit according to the manufacturer’s instructions (Qiagen, Germany). After digestion of genomic DNA, RNA samples were examined by PCR to ensure no DNA contamination was presented and then quantified by ND-1000 UV–Vis Spectrophotometer (ThermoFisher Scientific, USA). For reverse-transcription, purified RNA was converted to cDNA with random hexameters primer using the SuperScript First-Strand Synthesis (Invitrogen, USA) according to the manufacturer’s protocol. The cDNA samples were quantified using a Qubit 2.0 Fluorometer (Invitrogen, Carlsbad, CA) and cDNA quality was checked on a 0.8% agarose gel. The resulting RNA and cDNA were stored at − 80 °C until further use.

### Characterization of bacterial community

16S rRNA sequencing library was constructed using a MetaVx™ Library Preparation kit (GENEWIZ, Inc., South Plainfield, NJ, USA) which contains a panel of proprietary primers. Briefly, ~ 100 ng cDNA was used to generate amplicons that cover V3, V4 hypervariable regions of bacterial 16 S rDNA. Indexed adapters were added to the ends of the 16 S rDNA amplicons by limited-cycle PCR. DNA libraries were validated using an Agilent 2100 Bioanalyzer (Agilent Technologies, Palo Alto, CA, USA), and quantified by Qubit and real-time PCR (Applied Biosystems, Carlsbad, CA, USA). Libraries were multiplexed and loaded on an Illumina MiSeq instrument according to manufacturer’s instructions (Illumina, San Diego, CA, USA). Sequencing was performed using a 2 × 300 paired-end configuration; image analysis and base calling were conducted by the MiSeq Control Software on the MiSeq instrument. Demultiplexed 16 S rRNA gene raw reads and respective metadata are available in the NCBI Sequence Read Archive (SRA) database under BioProject PRJNA402085.

Demultiplexed paired-end Illumina reads from 51 samples (8 samples of TA and PA, TH and PH, TAH and PAH, TC and PC for each species of coral with three repeats, totally 48 samples for two corals, and 1 ambient seawater sample with three repeats) were subjected to quality and length trimming using Trimmomatic v.0.33 [29] using the threshold: base quality score ≥ 25 and sequence length ≥ 150 bp. After that, the PE reads were merged and screened (400 bp ≤ length ≤ 480 bp, allowing no ambiguous base and homopolymers < 8) by Mothur’s subroutines [30].

The QIIME 1.9 SOP [31] was used for the following analysis: (1) Sequences were clustered into operational taxonomic units (OTUs) at 97% similarity using open-reference OTU picking strategy by UCLUST; (2) representative sequences for each OTU were selected and aligned to the Greengene database by PyNAST; (3) chimeras were checked and filtered by ChimeraSlayer; (4) OTUs were taxonomically classified using RDP Naïve Bayesian classifier; (5) sequences from the OTUs that were classified as Mitochondria, or Chloroplast were filtered out (6) only the OTU with the observation count across all samples more than 50 were retained. The OTU abundance data were Hellinger-transformed and the similarities were displayed with nonmetric multidimensional scaling (nMDS) with Bray–Curtis distances.

### Metatranscriptome analysis of microbial functional response

RNA sequencing library was constructed using a ribosomal RNA depletion strategy. Briefly, rRNA from eukaryote or prokaryote were subtracted from total RNA by Ribo-zero rRNA removal kits (Epicenter Biotechnologies) and the rRNA-depleted mRNA was then fragmented for library construction with an estimated mean insert size of 300 bp. Metatranscriptome sequencing was carried out on Illumina HiSeq2500 platform using a 2 × 125 paired-end (PE) configuration.

The raw Illumina reads were trimmed using a minimum phred score of 20, a minimum length of 75 by Trimmomatic 0.33, allowing no ambiguous nucleotides and trimming off Illumina sequencing adaptors if found. For paired-end reads, only the forward sequences were submitted to MG-RAST server [32] for further processing and annotation using the standard pipeline (MG-RAST Project ID: *A. valida*, 18,173; *G. fascicularis*, 17,762). Taxonomic assignment of metatranscriptomic sequences was performed by metagenomics rapid annotation using subsystem technology (MG-RAST) using the M5NR database (BLAT, minimum identity ≥ 60%, minimum alignment length ≥ 15aa, e-value ≤ 1e−5). Sequences identified as bacteria were further annotated based on SEED subsystems (BLAT, minimum identity ≥ 60%, minimum alignment length ≥ 15aa, e-value ≤ 1e−5).

The matrix of functional abundance profile for the metatranscriptome was retrieved by MG-RAST API [33] and loaded into DESeq2 package Version 1.14.1 [34] for differentially expressed gene (DEG) analyses after normalization by rlong transform function. Features (genes) identified as differentially expressed were then corrected for false positives using the Benjamini and Hochberg false discovery rate (FDR) correction for multiple testing. A feature was considered significantly differentially expressed if it had an average basemean expression > 100 and it had an FDR adjusted *p* < 0.05. A total of seven pairwise comparisons were performed on the dataset to investigate differences in gene expression patterns in response to temperature and pH either in exposure or recovery set for each coral: (1) TA versus TC; (2) TH versus TC; (3) TAH versus TC; (4) PC versus TC; (5) PA versus PC; (6) PH versus PC; (7) PAH versus PC.

### Statistical analysis

For alpha and beta diversity analyses, data from each coral were analyzed separately and rarefied to the smallest sequencing depth from each dataset. Alpha diversity statistics including observed species, Chao1 richness estimations, Shannon, phylogenetic diversity (PD) whole tree [35] were calculated by QIIME and tested with analysis of variance (ANOVA) and further pairwise comparisons with the Tukey honest significant difference (HSD) test at 95% confidence level. Community similarity (beta-diversity) was visualized by nonmetric multidimensional scaling (nMDS) using the Bray-Curtis distance metric after Hellinger standardization. Analysis of similarities (ANOSIM) was used to test for significant differences between treatments. The R-statistic reported by ANOSIM is based on the difference of mean dissimilarity ranks between groups and within groups and ranges from 0 (no separation) and 1 (complete separation). The homogeneity of multivariate dispersions was tested using PERMDISP (permutation of dispersion), followed by permutational multivariate analysis of variance (PERMANOVA, adonis in vegan package), which determined whether the grouping of samples by treatment is statistically significant. Community dissimilarity between treatment groups and control groups were calculated by similarity percentages (SIMPER), which provides information about how different the communities are, and reports what specific OTUs are driving those differences. SIMPER was performed to examine which OTUs contributed most to the dissimilarity between treatments. PCoA was conducted according to the matrix of distance. Correlation between variables was computed using Spearman rank correlation, and a scatter plot was generated using R package “ggplot2”. All statistical analyses were carried out in R using vegan package [36]. All statistical tests were performed in R (version 2.15.3 or later). Unless otherwise stated, data are presented as means ± standard errors of the means (SEM).

## Results

### In situ active coral microbial community change under/after (6/9 d) A, H and AH stressors

A total of 2,024,156 high-quality 16S rRNA pyrosequencing reads were recovered from 51 libraries of the total RNA of two species of corals and the ambient seawater with three repeats, respectively. At the OTU level, PCoA plot illustrated the distinct prokaryotic symbionts of the two coral species, which clustered together apart from the seawater (Additional file 1: Fig. S1), indicating that coral host specificity plays the major role in shaping the microbial communities rather than environmental factors.

**Fig. 2:**
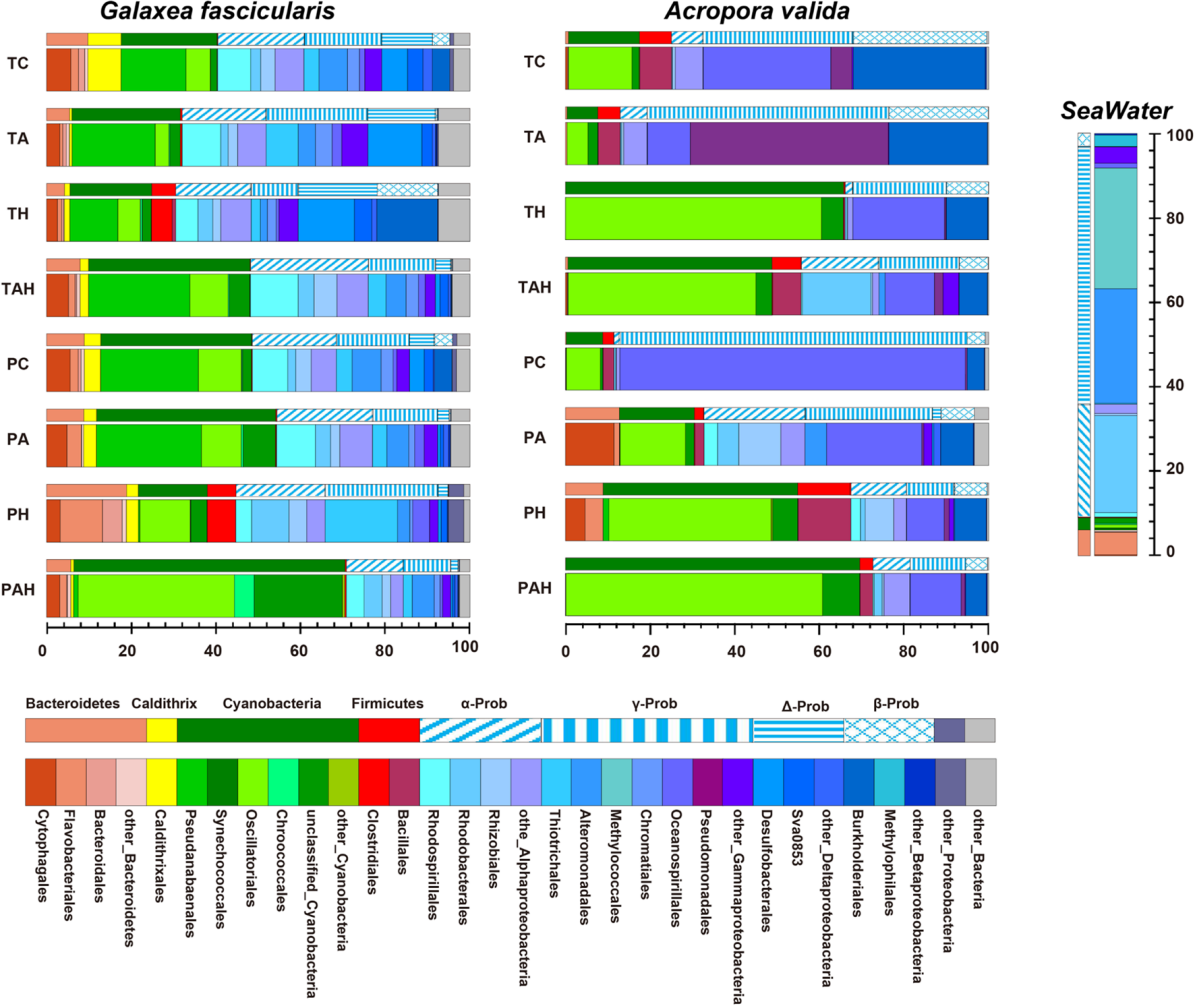
Relative abundance of the bacterial community in *Galaxea fascicularis* and *Acropora valida* under different treatments at phylum (Proteobacteria at the class level) and order levels. T: under stress; P: after stress; A: acidification; H: warming; AH: acidification–warming

Based on a similarity level of 97%, a total of 1443 operational taxonomic units (OTUs) were resolved: *G. fascicularis* 1403 OTUs, *A. valida* 473 OTUs and seawater 248 OTUs. *G. fascicularis* hosted higher bacterial diversity than *A. valida* in all treatment groups (Table 1, Additional file 1: Table S2; Fig. 2). Based on the alpha diversity analysis using phylogenetic diversity (PD) (Additional file 1: Table S2), PD score of *G. fascicularis* was highest in the TA group (112.81 ± 3.44) and TH showed the lowest score (94.26 ± 6.43). Among all groups, no significant differences (ANOVA, *P* > 0.05) were detected in comparison with their corresponding control (TC or PC). For *A. valida*, significant increases of microbial alpha diversity were observed in TAH, PA, and PH from *A. valida* comparing to their control group (TC or PC). TAH group (PD = 33.73 ± 3.4) showed the highest bacterial alpha diversity in comparison with TC (PD = 24.63 ± 1.14; ANOVA, *P* < 0.05). The PD values in PA groups (PD = 50.67 ± 1.92, ANOVA, *P* < 0.05) and PH (41.21 ± 8.75, ANOVA, *P* < 0.05) were significantly higher than that in PC group.

**Table 1:** Adonis (PERMANOVA) results testing differences in taxonomic composition (a) and functional composition (b) between different treated groups and their corresponding controls (TC or PC) based on Bray–Curtis distances

*G. fascicularis*	*d.f.*	*SS*	*MS*	*Pseudo-F *	*R*^*2*^	*P*_*perm*_^*ab*^	*A. valida*	*d.f.*	*SS*	*MS*	*Pseudo-F *	*R*^*2*^	*P*_*perm*_^*ab*^
*a*													
TA vs. TC	1	0.218	0.218	4.553	0.07	0.069.	TA vs. TC	1	0.364	0.364	7.888	0.10	0.022*
TH vs. TC	1	0.402	0.402	8.410	0.14	0.004**	TH vs. TC	1	0.370	0.370	8.036	0.10	0.015*
TAH vs. TC	1	0.214	0.214	4.481	0.07	0.081.	TAH vs. TC	1	0.387	0.387	8.395	0.11	0.009**
PC vs. TC	1	0.133	0.133	2.775	0.04	0.142	PC vs. TC	1	0.217	0.217	4.699	0.06	0.043*
PA vs. PC	1	0.308	0.308	6.440	0.10	0.114	PA vs. PC	1	0.252	0.252	5.472	0.07	0.031*
PH vs. PC	1	0.574	0.574	12.010	0.19	0.001***	PH vs. PC	1	0.409	0.409	8.868	0.11	0.003**
PAH vs. PC	1	0.355	0.355	7.421	0.12	0.001***	PAH vs. PC	1	0.913	0.913	19.818	0.25	0.001***
Residuals	16	0.765	0.048		0.26		Residuals	16	0.737	0.046		0.20	
Total	23	2.970			1.00		Total	23	3.649			1.00	
*b*													
TA vs. TC	1	0.024	0.024	4.806	0.10	0.001***	TA vs. TC	1	0.008	0.008	2.756	0.03	0.292
TH vs. TC	1	0.022	0.022	4.452	0.09158	0.003**	TH vs. TC	1	0.019	0.019	6.389	0.08	0.007**
TAH vs. TC	1	0.025	0.025	5.046	0.10	0.001***	TAH vs. TC	1	0.077	0.077	25.354	0.31	0.001***
PC vs. TC	1	0.013	0.013	2.640	0.05431	0.126	PC vs. TC	1	0.016	0.016	5.379	0.07	0.094.
PA vs. PC	1	0.016	0.016	3.277	0.06742	0.082.	PA vs. PC	1	0.016	0.016	5.371	0.07	0.098.
PH vs. PC	1	0.041	0.041	8.333	0.17142	0.001***	PH vs. PC	1	0.019	0.019	6.253	0.08	0.002**
PAH vs. PC	1	0.020	0.020	4.056	0.08344	0.078.	PAH vs. PC	1	0.046	0.046	14.989	0.18	0.001***
Residuals	16	0.080	0.005		0.32915		Residuals	16	0.049	0.003		0.19	
Total	23	0.242			1.00		Total	23	0.251			1.00	

*d.f.*, degree of freedom; *SS*, sum of squares; *MS*, mean sum of squares.

^a^ Significance value based on 999 permutations. ^b^ Significance level: .<0.1, *< 0.05, **< 0.01, ***<0.001. T: under stress; P: after stress; A: acidification; H: warming; AH: acidification–warming

To determine the response patterns of the coral prokaryotic symbionts, the OTU abundance data were Hellinger-transformed and the similarities were displayed with nonmetric multidimensional scaling (nMDS) with Bray–Curtis distances. At the OTU level (97% sequence similarity), clear differences in community composition were observed across different tested groups in both *G. fascicularis* (ANOSIM; R = 0.86, *P*-value = 0.001) and *A. valida* (ANOSIM; R = 0.89, *P*-value = 0.001) (Additional file 1: Fig. S2). Homogeneous multivariate dispersion among sampling groups showed no significant difference in both corals (PERMDISP, *P* > 0.05) and allowed us to compare the community structure between treated groups and their corresponding control groups (TC or PC) using PERMANOVA with customized contrasts (Table 1). Community dissimilarity between treatment groups and control groups were calculated by similarity percentages (SIMPER), which provides information about what specific OTUs are driving those differences.

**Fig. 3:**
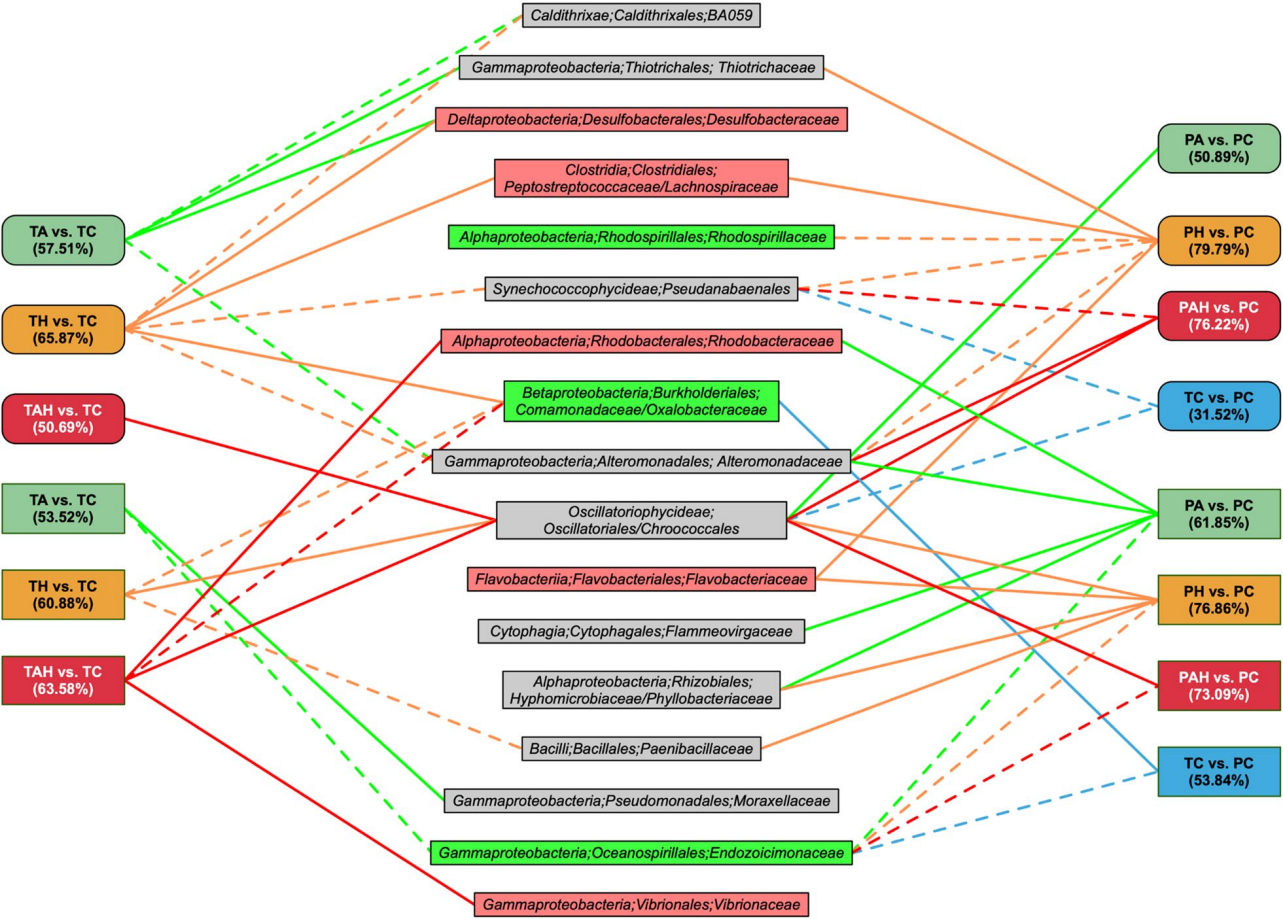
Schematic diagram shows prokaryotic symbionts causing most of the divergence in community composition throughout the stress experiment of *G. fascicularis* (Rounded rectangle) and *A. valida* (Rectangle) determined by Similarity Percentages (SIMPER) analysis. Solid line showed increased relative abundance of given prokaryotic families and dash line represent decreased comparing to control groups (TC or PC). Number in parentheses indicates overall dissimilarity, and only the most significant families (contribution ≥ 2%) driving differences between the stress group and control group were shown. The microorganisms in the green box represent common microorganisms in healthy corals, and the red box represent coral bleaching and disease related. T: under stress; P: after stress; A: acidification; H: warming; AH: acidification–warming

The bacterial community with transcriptional activity of massive coral *G. fascicularis* was compose of 29 phyla, and the most abundant phylum was Proteobacteria (Fig. 2). The most abundance class was Alphaproteobacteria (13.5–27.8%) followed by Gammaproteobacteria (11.2–26.7%), the other abundant bacteria were Cyanobacteria (16.3–64.1%), Bacteroidetes (4.2–18.9%), Caldithrix (0.6–7.9%) and Firmicutes (0.1–6.8%). Significant differences in community composition were observed in the warming (H) treatment (TH vs. TC/PH vs. PC; PERMANOVA, *P* < 0.05) (Table 1). SIMPER analyses indicated that the increased OTUs were affiliated with *Desulfobacteraceae*, *Burkholderiales*, *Clostridiales* and the decreased were *Pseudanabaenales*, *Caldithrixales*, *Alteromonadaceae* which were the primary drivers of difference between TH and TC (Fig. 3). Even after H stress, coral microbial structure change still continued. The increased OTUs were affiliated with *Thiotrichales*, *Flavobacteriales*, *Clostridiales* and the decreased OTUs were affiliated with *Pseudanabaenales*, *Rhodospirillales*, *Alteromonadales* (PH vs. PC). Although the overall microbial community composition was little affected by AH (TAH vs. TC; PERMANOVA, *P* > 0.05), significant differences in community composition were observed after the AH stress (PAH vs. PC; PERMANOVA, *P* < 0.05) (Table 1a), e.g. increased *Chroococcales, Oscillatoriales*, *Alteromonadales*, and decreased *Pseudanabaenales*, *Synchococcophycideae* (Fig. 3).

The transcriptional active bacteria in branching coral *A. valida* included 17 phyla, similar as *G. fascicularis*, the most abundant phylum was Proteobacteria (Fig. 2). In this phylum, the most abundance class was Gammaproteobacteria (11.2–82.1%) followed by Cyanobacteria (7.3–69.5%), Firmicutes (0.4–12.6%) and Bacteroidetes (0.1–8.9%). Unlike *G. fascicularis*, SIMPER analysis demonstrated that the OTUs affiliated with *Endozoicimonaceae* and *Comamonadaceae* were the major drivers of differences between the two control groups (TC vs. PC; PERMANOVA, *P* < 0.05; Table 1a), which were removed in the following diversity comparison. Significant differences in microbial community composition were caused by AH followed by H and A and these changes still remained after the stress removal (Table 1a). SIMPER analysis indicated that the increased OTUs were affiliated with *Moraxellaceae*, *Pseudomonadales*, *Oscillatoriophycideae*, *Chroacoccales*, *Vibrionales* and the decreased relative abundance of OTUs affiliated with *Endozoicimonaceae*, *Oceanospinileles*, *Paenibacillaeae*, and *Bacillales* were the primary drivers of difference (Fig. 3). Meanwhile after stress removal, the increased OTUs were affiliated with *Flammeovirgaceae*, *Hyphomicrobiaceae*, *Rhizobiale*s, *Rhodobacteraleae*, *Alteromonadaceae* and the decreased OTUs were *Endozoicimonaceae* and *Oceanospinileles* (Fig. 3). Particularly, *Oscillatoriales* was dominant in TH and TAH groups (relative abundance up to 58.6% in TH and 42.6% in TAH) compared with that in TC (11.5%), and still maintained majority (38.3% in PH, 60.6% in PAH) after the stress removal.

In summary, A, H and AH stressors are able to change the community structure and relative abundance of microbes with transcriptomic activity. Under these stressors the bacterial community shifts away from typical coral microbial assemblages towards more opportunistic and potentially pathogenic communities because some potential pathogenic bacteria abundance increased (Figs. 2 and 3). Even after theses stresses were removed, these changes still maintained. Totally, the order of influence on microbial community structure is AH > H > A for *A. valida*, and H > A > AH for *G. fascicularis* based on Table 1a. *A. valida* prokaryotic symbionts is much more sensitive than *G. fascicularis*. Compared with A and H, AH showed synergistic acceleration effect on *A. valida* prokaryotic symbionts, while antagonistic effect on *G. fascicularis* prokaryotic symbionts since the change of TAH versus TC showed no significant difference, while TH vs. TC was significantly different (Table 1).

### Coral microbial gene expression change under/after (6/9 d) A, H and AH stressors

**Fig. 4:**
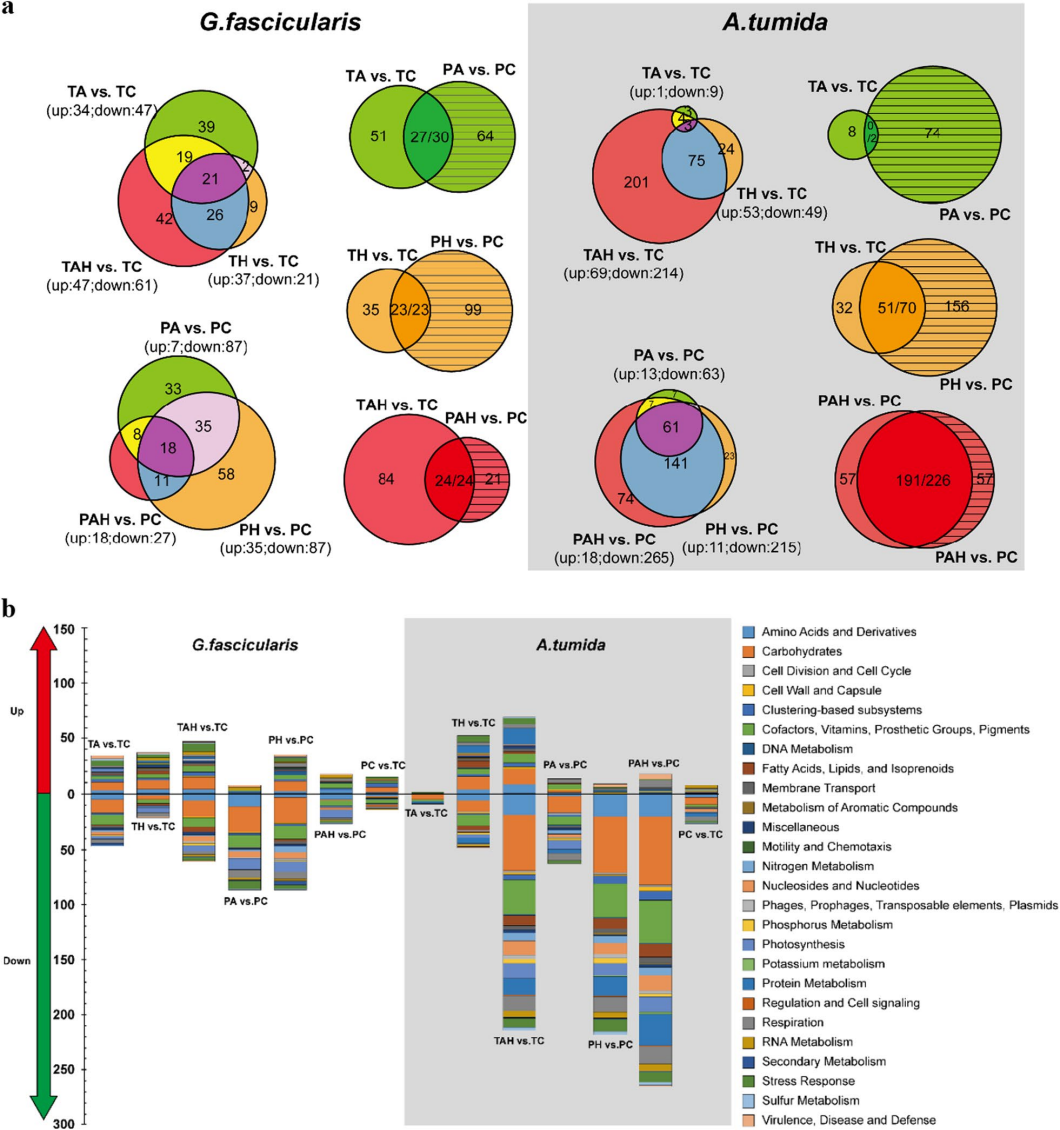
The DEGs analysis under (T) and after (P) warming (H), acidification (A) and warming-acidificaion (AH). **a** Venn diagram indicates the total DEGs number from each group and the overlapping areas based on and sampling time form each coral. The number separated by/indicates consistent expressed and total DGEs between T and P within some treatment. **b** Summary of the DGEs in each treatment. Genes are categorized by the level 1of the SEED Subsystems. T: under stress; P: after stress; A: acidification; H: warming; AH: acidification–warming

A total of 1,385,967,802 Illumina reads (*G. fascicularis* 24,312,711 ± 2,196,775 reads, *A. valida* 23,435,947 ± 1,282,676 reads) recovered from 48 metatranscriptomes (8 samples of TA/PA, TH/PH, TAH/PAH, TC/PC for each coral with three repeats respectively) were submitted to MG-RAST sever (Metagenomic Rapid Annotation using Subsystem Technology) for further annotation after quality control. According to MG-RAST pipeline, a mean of 17,174,699 ± 1,223,688 and 14,266,010 ± 837,839 reads containing predicted proteins with known functions were obtained from *G. fascicularis* and *A. valida*, respectively. After the selection based on taxonomic classification, prokaryotic transcripts were functionally annotated based on SEED subsystems. A total of 6145 and 4,660 genes with transcriptomic activity were identified from *G. fascicularis* and *A. valida*, respectively. These genes were classified into 28 categories. The most abundant categories were: Carbohydrates (21.4%), Photosynthesis (17.5%) and Amino Acids and Derivatives (8.7%) for *G. fascicularis*; Carbohydrates (22.7%), Amino Acids and Derivatives (10.0%) and Photosynthesis (7.4%) for *A. valida.* To further interrogate the effects of stresses on coral microbial functional properties, we conducted a differentially expression genes (DEGs) analysis. In total, 273 and 374 genes were differentially expressed in *G. fascicularis* and *A. valida*, respectively (Fig. 4), indicating the impacts of these stressors on coral prokaryotic symbionts functions (Additional file 1: Fig. S2). Considering the possible perturbation during the experiment, DEGs from the comparisons of two control groups (TC vs. PC) were discarded. 251 and 369 DEGs from *G. fascicularis* and *A. valida* respectively were used for following analysis.

In the case of massive *G. fascicularis*, DEGs had an average fold change of 2.02 (range, 1.21–21.49) for up-regulated genes and − 1.60 (range, − 1.21 to − 4.20) for down-regulated genes. The most DEGs were found in TAH versus TC comparison (47 up, 61 down), suggesting AH has the most important impact on *G. fascicularis* microbial function which was different from H which had the most impact on *G. fascicularis* microbial diversity, followed by A (TA vs. TC:34 up, 47 down), and H (TH vs. TC: 37 up, 21 down) (Fig. 4). Under AH, the most highly up-regulated gene (21.49-fold increase) was L-alanine glyoxylate aminotransferase (EC 2.6.1.44), which is involved in photorespiration, The most highly down-regulated gene (4.20-fold reduction) was a Cytosol aminopeptidase PepA (EC 3.4.11.1) involved in the processing and regular turnover of intracellular proteins. Besides, genes involved in essential amino acids biosynthesis (lysine and methionine), respiration, photosynthesis (expect *PsaB*, *PsbB*, and *PsbD* in TA), Calvin–Benson cycle, secondary metabolism (auxin biosynthesis), and related genes were also down-regulated expressed. Genes related to stress response (such as molecular chaperones, ROS antioxidants, DNA repair related genes), adhesion (such as fibrinogen binding protein, cell wall anchored protein SasA) were significantly up-regulated expressed. After removing these stressors, the most DEGs were found in PH vs. PC comparison (35 up, 87 down), followed by the PA vs. PC comparison (17 up, 87 down) and PAH vs. PC comparison (18 up, 27 down) (Fig. 4). This means that, from a long-time perspective, most of the DEGs caused by AH could return to their original expression level after stress removal. Particularly, we found some new emerging DEGs after stress removal, e.g. the down-regulated of *PsbA*, *PsbB* after A stress removal and the up-regulated of adhesion related gene fibrinogen-binding protein after H stress removal.

For branching *A. valida*, DEGs had an average fold change of 1.92 (range, 1.28–30.41) for up-regulated and − 2.25 (range, − 1.27 to − 14.34) for down-regulated genes. The most DEGs were found under combined AH stress (TAH vs. TC: 69 up, 214 down), followed by H (TH vs. TC: 53 up, 49 down) (Fig. 4). The most highly up-regulated gene (30.41-fold increase) was D-amino-acid oxidase (DAO, EC 1.4.3.3) under AH stress, which can regulate host innate immune factor that modulates the growth of both pathogens and commensals in gut [37]. The most highly down-regulated gene (8.75-fold reduction) was Photosystem II CP43 protein (PsbC) gene under AH stress. When these stresses were removed, many DEGs e.g. genes involved in folate, pterine and tetrapyrroles biosynthesis under H and AH were found in PAH vs. PC comparison (18 up, 265 down), followed by PH vs. PC comparison (11 up, 215 down) (Fig. 4), indicating the carryover effect of AH and H on *A. valida* microbial metabolism. Some new emerging DEGs included down-expressed Calvin Benson cycle and photosynthesis related genes when H stress removal, and the up-regulated genes associated with antibiotics and toxic compounds resistance protein czcA, cusA and cmeB when AH stress removal.

**Fig. 5:**
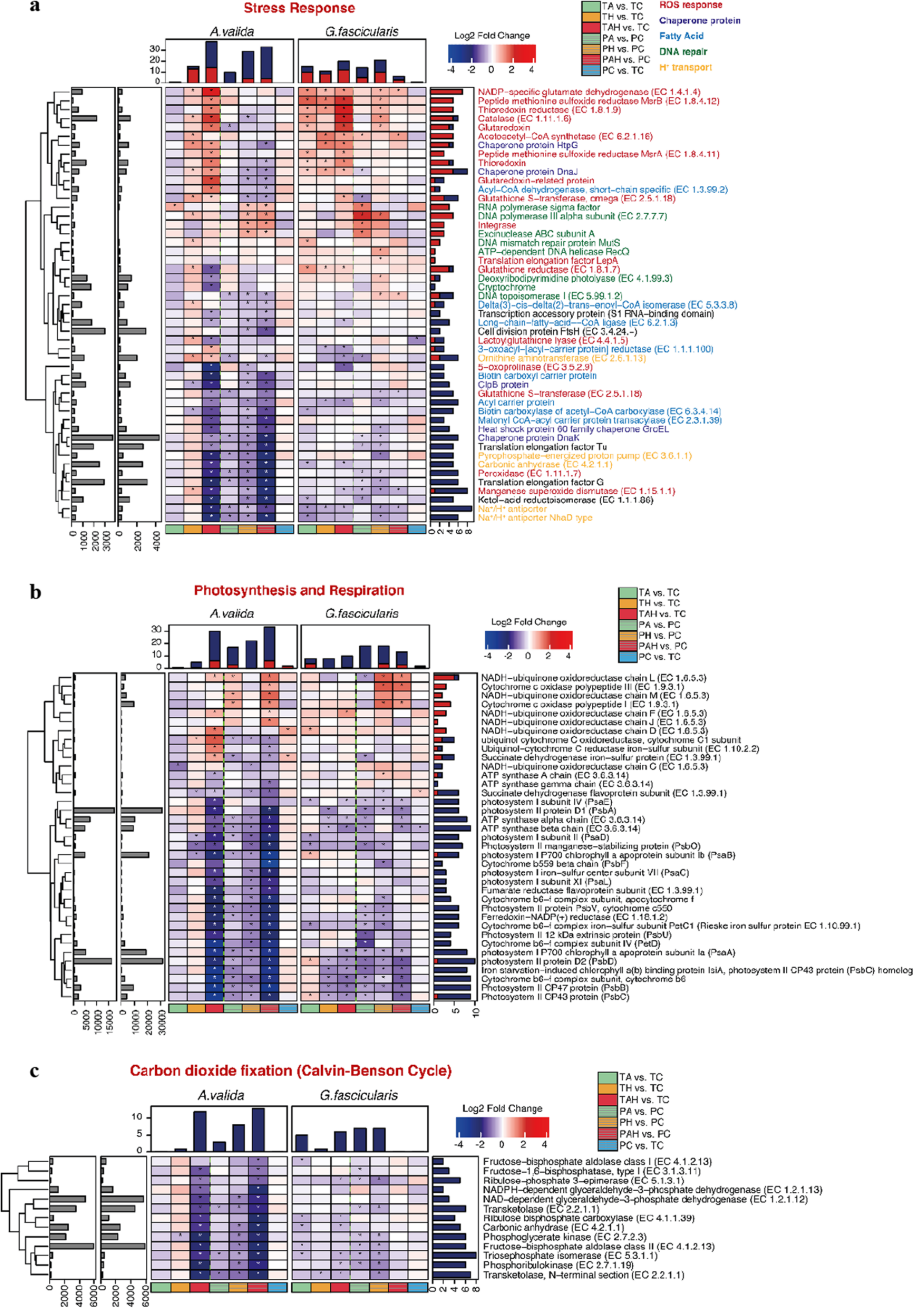

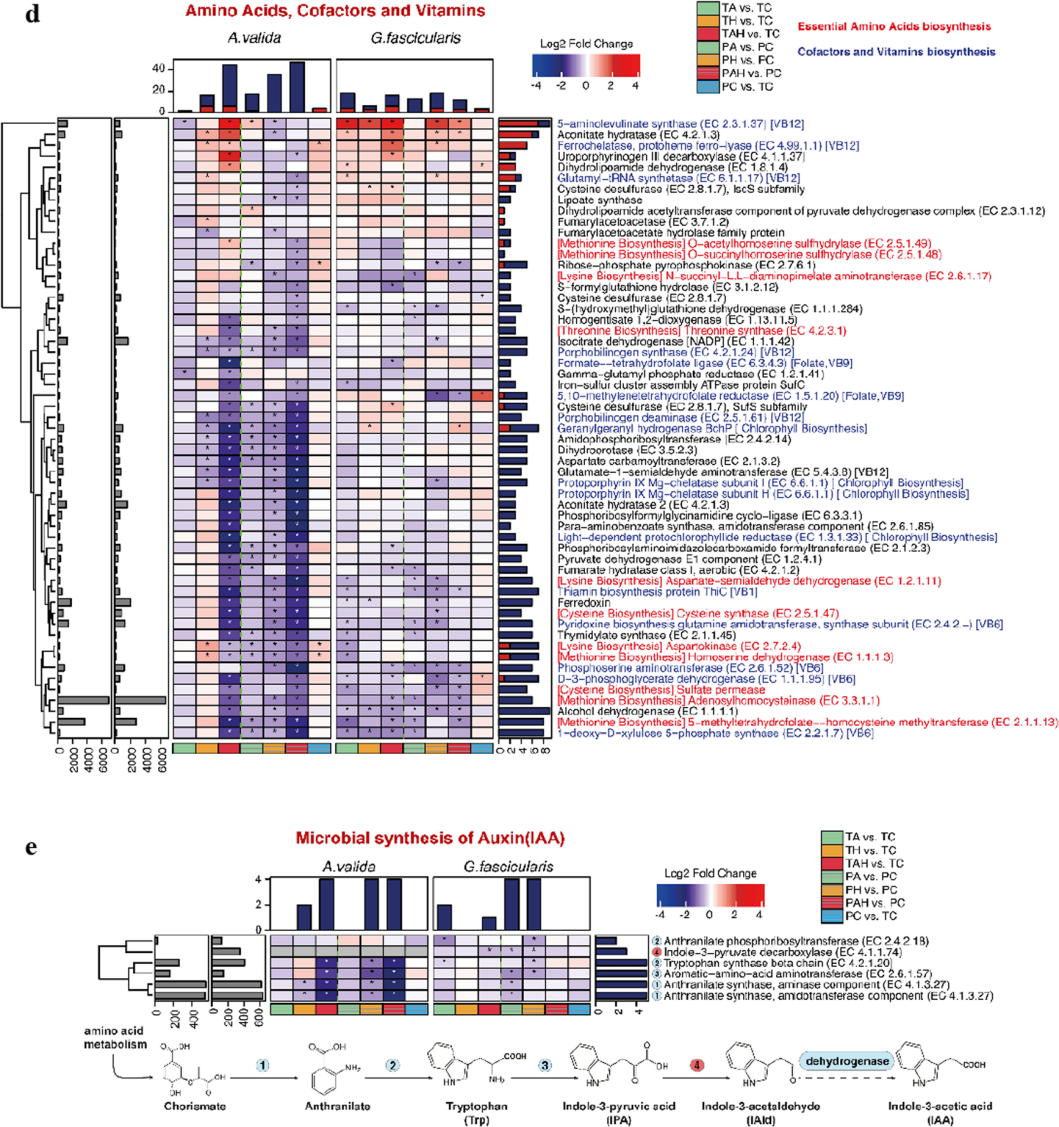
Gene expression changes based on the DEGs analysis. **a** stress response; **b** photosynthesis and respiration; **c** carbon dioxide fixation; **d** amino acids, cofactors and vitamins; f: auxin. T: under stress; P: after stress; A: acidification; H: warming; AH: acidification–warming

Based on the DEGs comparison between the two species of corals, the number DEGs in *A. valida* were much more than *G. fascicularis* (Figs. 4 and 5) indicating their different sensitivity to these stresses. Based on DEGs, the order of influence on microbial gene expression is AH > H > A for *A. valida*, and AH > A > H for *G. fascicularis*, respectively. Compared with A and H, AH showed synergistic acceleration effect on *A. valida* and *G. fascicularis* microbial gene expression since both corals had the most DEGs under AH (Fig. 4). The interactive effect from A and H is also supported by Fig. S3, DEGs patterns shifted obviously under AH stress compared with single A or H stress.

**Fig. 6:**
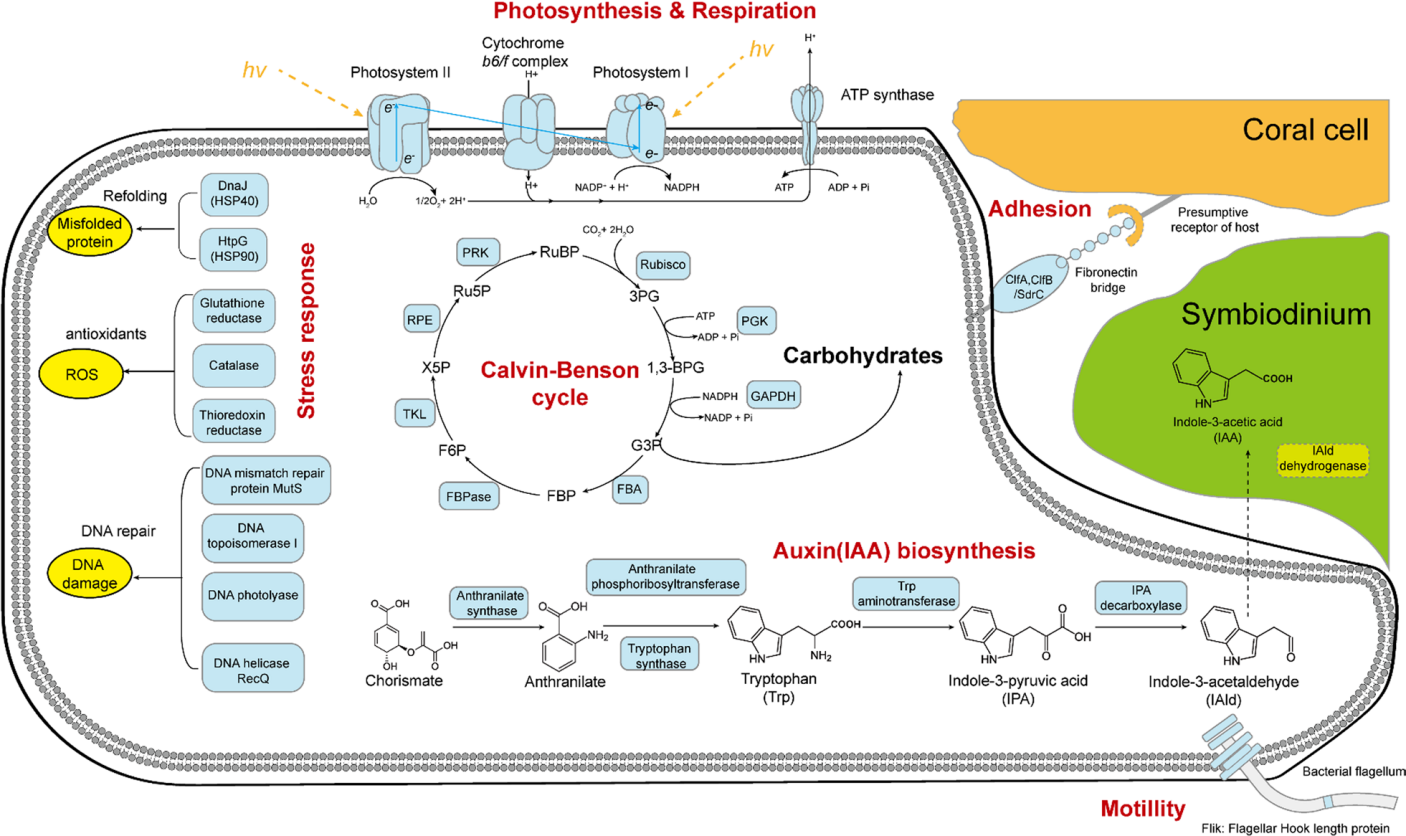
The DEGs involved in the metabolism of coral prokaryotic symbionts

Taking together (Fig. 6), functional genes involved in Carbohydrates, Cofactors, Vitamins, Prosthetic groups, Pigments, Protein metabolism, Amino acids and derivatives, and Stress response Genes involved in essential amino acids biosynthesis (cysteine, lysine and methionine), photosynthesis, Calvin–Benson cycle and folate/pterines metabolism were down-regulated; genes involved in stress response (such as chaperone proteins, antioxidants) and bacterial signal recognition particle (*Ffh*, *FtsY* and *SecY*) were up-regulated. These results indicated impacts of these stressors on the metabolism of coral holobionts in spite of coral species.

## Discussion

### A, H and AH stressors change coral’s in situ active microbial community towards potentially pathogenic bacteria

Taxonomic shifts of microbes of corals under environmental stress have been observed [17–20, 24, 38–40]. Corals in lowered pH had higher microbial diversity compared with the control [41]. Higher bacterial diversity was represented in bleached sample compared to health sample during a bleaching event [19]. Such changes could result in the disequilibrium of the coral microbial community structure. Compared with the study using 16 S rDNA, 16 S rRNA could reflect exactly the ‘active’ fraction of microbial community. In this study, using a RNA-based sequencing approach we were able to detect significant differences in the in situ prokaryotic symbionts under different stresses.

Vezzulli et al. [42] reported that destabilization of the coral holobionts was concomitant with a microbial community shift towards opportunistic microorganisms or potential pathogens, such as *Vibrio* spp. due to thermal stress. In this study, based on RNA-based diversity analysis of coral prokaryotic symbionts, coral *G. fascicularis* hosted a significantly higher relative abundance of Desufobacterales in the thermal- or acidified groups. Besides, some potentially coral pathogens, e.g. Clostridiales, *Vibrionaceae*, *Flavobateriaceae*, *Rhodobacteraceae*, and *Desulfobacteraceae* were found in the corals under stress. Particularly significant increase of *Rhodobacteraceae* and *Vibrionaceae* in *A. valida* under AH stress was detected.

Besides the diversity change, another primary driver of microbial variation was the relative abundance change, for example reduced relative abundance of *Caldithrixales, Pseudanabaenaceae* (*Cyanobacteria; Synechococcophycideae*) in *G. fascicularis*, putatively endosymbiotic *Endozoicimonaceae* in *A. valida*, and increased abundance of *Oscillatoriales* (*Cyanobacteria*; *Oscillatoriophycideae*) for both coral species were detected in this study. Compared with the control, coral *A. valida* showed significant higher relative abundance of Cyanobacteria in all H and AH groups, the relative abundance of *Chroococcales* and *Oscillatoriales* increased in both bleached corals, indicating Cyanobacteria’s replacement potential for Symbiodiniaceae under stress. This is probably a strategy for coral holobionts try to maintain the carbon utilization from destroyed photosynthesis of Symbiodiniaceae.

Based on our results, coral species-dependent microbial community change with thermal/acidification stress was suggested. Though A, H and AH stressors could change both corals’ in situ active microbial diversity, H showed most negative effects on *G. fascicularis* microbial composition, while H, AH on *A. valida* microbial composition (Table 1a, Additional file 1: Table S2). The DEGs number in *A. valida*. was bigger than *G. fascicularis* and most of the DEGs were down-regulated in *A. valida*. Totally *A. valida* prokaryotic symbionts was much more sensitive than *G. fascicularis* prokaryotic symbionts, indicating coral-species dependent response and recover patterns (Table 1; Additional file 1: Table S1; Figs. 2 and 3). Based on the weighted unifrac distance analysis, although the microbial community structure of *G. fascicularis* was not completely recovered when the stress was removed, its recoverability was better than that of *A. valida* (avg. weighted unifrac distance: 0.360–0.651 vs. 0.607–0.876). Accordingly, we hypothesized that coral species with higher microbial diversity may have a greater probability of the presence of stress-resistant taxa or more complex interaction-network and develop functional redundancy that could maintain stability of community structure and even confer stress tolerance to their holobionts. This hypothesis is in consist with the research of Yu et al. [43], they also suggested that the higher tolerance of *Pavona decussata* compared with that of *Acropora pruinosa* might result from a complex biological process caused by higher symbiotic bacterial diversity, different dominant bacteria, higher host immune and stress resistance responses, and lower metabolic rate.

Recently study showed that bacterial diversities associated with massive coral were generally higher than those associated with branching corals [44]. The thickness of tissue layer influences the ability of microbes to colonize [45], *G. fascicularis* with a thicker tissue may provide a higher complex niche for microbial colonization which is supported by the fact that massive *G. fascicularis* hosted higher bacterial diversity than *A. valida* in all treatment and control groups. Interestingly, OTUs affiliated with *Caldithrix* were only detectable in the prokaryotic symbionts of *G. fascicularis*. This bacterial phylum has been recently recognized as a new independent phylum-level clade, and were identified from the sponges [46], ascidians [47] and marine hydrothermal vents [48]. Genomic analysis of the representative lineage *Caldithrix abyssi*, demonstrated the ability to synthesize nucleotides, most amino acids and vitamins as well as contain genes encoding proteins that confer O_2_ tolerance, suggested that such a flexible metabolism has the potential to help *C. abyssi* to adapt to changing conditions [48]. The results from Grottoli et al. [49] also indicted that temperature-stress tolerant corals have a more stable microbiome and propose that coral with a stable microbiome were also more physiologically resilient and thus more likely to persist in the future. Ocean acidification as a result of increased anthropogenic CO_2_ can cause a shift in coral-associated microbial communities of *p*CO_2_-sensitive corals. But in the case of *p*CO_2_-tolerant coral, massive *Porites* spp. showed a high degree of tolerance to OA [50].

### A, H and AH stressors change the coral microbial metabolism and destabilize the coral-microbes symbioses

Thurber et al. [51] evaluated the structural and functional changes in coral microbial communities of *Porites compressa* under increased temperature, reduced pH stressors. They found that stressors increased the abundance of microbial genes involved in virulence, stress resistance, sulfur and nitrogen metabolism, motility and chemotaxis, fatty acid and lipid utilization, and secondary metabolism. Ziegler et al. [22] found that functional profiles of microbial communities changed with thermal stress, several functions related to carbohydrate metabolism were enriched. The study of Rädecker et al. [52] also indicated that heat stress destabilizes symbiotic nutrient cycling in corals, which will reduce the coral holobiont health. Ocean acidification could result in the increase of virulence-associated gene expression and shifts in the community composition, e.g. increases in opportunistic pathogens such as *Vibrionaceae* and *Alteromonadaceae* and a loss of putatively symbiotic *Endozoicomonas* spp. [43]. Thus, the diversity and relative abundance changes of coral microbiota caused by stress could alter microbial metabolism, profoundly shift the health status of the coral holobionts.

In this study, H and AH stressors could change both corals microbial gene expression obviously (Table 1b; Figs. 3, 4 and 5). For both corals tested, the DEGs mainly include Carbohydrates, Cofactors, Vitamins, Prosthetic Groups, Pigments, Protein Metabolism, Amino Acids and Derivatives, and Stress Response, indicating the broad influences of A, H and AH stressors on coral microbial metabolisms. Particularly, functional gene analysis demonstrated that stressors increased the abundance of microbial genes involved in virulence, stress resistance, sulfur and nitrogen metabolism, motility and chemotaxis, fatty acid and lipid utilization, and secondary metabolism. For example, *Vibrionaceae, Flavobateriaceae, Rhodobacteraceae*, and *Desulfobacteraceae* showed up-regulated expression patterns (Fig. 3). In addition, persistent effect on coral microbial metabolisms was also suggested even after the stress was removed for 9 d, since some DEGs exhibited in exposure groups were difficult to recover to that of the control level, especially after H stress for *G. fascicularis* and AH, H for *A. valida*. Meanwhile, a broad array of new DEGs emerged after the stress, suggesting a continued effect on the coral microbial metabolisms.

A, H and AH show great impacts on coral-microbes symbioses since the expression of nutrient metabolism related genes’ expression was mostly down-regulated (Fig. 5), e.g. many DEGs involved in photosynthesis, carbon dioxide fixation, amino acids, cofactors and vitamins, auxin synthesis were detected), indicating the blocked microbial metabolism especially in branching *A. valida* (Fig. 5). Interestingly, plant hormone gene expression was also down-regulated under these stresses, suggesting the possible corrupted relationship of microbes with Symbiodiniaceae (Figs. 5 and 6). It is known that bacterial auxin can cause interference with plant developmental processes [53]. Indole-3-acetic acid (IAA), the major naturally occurring auxin, was detected in marine sediments, and was proposed producing by various marine bacteria, may affect algal growth in natural environments [54]. IAA synthesis related gene expression changes may affect the growth and reproduction of symbiotic algae, thus, affect the entire symbiotic system in corals, e.g. the interaction between bacteria and algae in corals could be reduced. In this study, microbial endemic gene IPA (iso-propyl alcohol) decarboxylase gene (the whole reaction rate-limiting step) was down-regulated in each treatment group. The bacterial auxin synthesis and adhesion genes were significantly down-regulated after stress removal.

### Synergetic interaction of combined A and H on the coral prokaryotic symbionts

Synergetic effects could either offset each other (i.e. antagonistic effect, where two stressors interact to produce an effect that one stressor reduces/mitigates the level of another) or aggravate it through an accumulation of stress (acceleration effect). Prada et al. [25] showed a synergistic adverse effect on the mortality rates of corals (*Balanophyllia europaea*, *Leptopsammia pruvoti*, and *Astroides calycularis*)(up to 60%), suggesting that high seawater temperatures may have increased their metabolic rates which, in conjunction with decreasing pH, could have led to rapid deterioration of cellular processes and performance. Pitts et al. [24] also suggested that ocean acidification partially mitigates the negative effects of warming on the larval development of *Orbicella faveolata*. Agostini et al. [26] investigated the effects of elevated temperature and high pCO_2_ on *G. fascicularis* with oxygen and pH microsensors and found that, under a combination of high temperature and high CO_2_, the photosynthetic rate increased to values close to those of the controls indicating an antagonistic effect on the photosynthesis of *G. fascicularis* holobiont. In the case of coral microbiome’s response to warming and acidification, Webster et al. [39] explored the microbiome response of corals e.g.* Acropora millepora*, *Seriatopora hystrix* to near-future climate change conditions. An interactive acceleration effect between stressors was also identified, with distinct communities developing under different pCO_2_ conditions only evident at 31 °C.

In this study, the interactive effect from A and H on coral prokaryotic symbionts was indicated by the comparison between combined AH and A or H alone. The DEGs patterns shifted the most obvious under AH stress based on the distribution of DEG fold change (Fig. S3). In the case of branching coral *A. valida*, AH showed acceleration effect on both the in situ active microbial community and gene expression since the microbial diversity and DEGs number were the highest under AH stress compared with A or H (Table 1; Figs. 4 and 5), and this acceleration effect from AH still existed when AH was removed. The acceleration effect of acidification and heat stress on *A. valida* prokaryotic symbionts in this study is consist with the suggestion from webster et al. [39].

For massive coral *G. fascicularis*, AH showed acceleration effect on microbial gene expression compared with single A or H stress because AH caused the most DEGs (Fig. 4), which was also supported by PERMANOVA analysis in Table 1 (*pseudo*-F 5.046 in AH treatment bigger than *pseudo*-F 4.806 in A treatment) (Fig. 4; Additional file 1: Fig. S3). But, the acceleration effect on microbial gene expression disappeared when AH was removed. Interestingly, AH showed antagonistic effect on microbial community structure in stress treatment (T) and removal groups (P) because A was able to mitigate the effect of H on the microbial community structure change. Different from *A. valida*, *G. fascicularis* recover better when AH stress was removed and the acceleration effect was not on-going. The different interactive effect of AH on coral *G. fascicularis* from *A. valida* indicates the coral species-dependent response to stress, which may result from their different microbial communities.

## Conclusion

Based on the RNA-based diversity and gene expression comparison in this study, A, H and AH stressors change coral’s in situ active microbial community structure and functional gene expression profiles, whatever during and after these stresses, which probably cause shifts in coral prokaryotic symbionts from healthy mutualistic relationships to those that are more pathogenic and detrimental to the coral host. DEGs comparison indicates that these stressors could increase the abundance of microbial genes involved in virulence, stress resistance, and heat shock proteins. Many DEGs involved in photosynthesis, carbon dioxide fixation, amino acids, cofactors and vitamins, auxin synthesis are down-regulated, indicating the destabilized or destroyed coral-microbes symbioses. In particular, compared with single stress, synergetic effects of combined AH on microbial diversity and gene expression are indicated. Massive *G. fascicularis* with higher microbial diversity shows more tolerance than branching *A. valida*, indicating coral species-dependent microbial response patterns.

Further physiological and biochemical studies are required to ascertain the consequences of these functional microbial diversity and gene expression shifts caused by acidification and /or warming since this study was only carried out using metatranscriptome strategy. The different response patterns of sensitive and tolerant corals indicated in this study highlight the importance of coral prokaryotic symbionts in assessing the impacts from thermal and/or acidification, which is helpful for us to predict the consequences of global climate change for coral reef ecosystems.

## Supplementary Information

**Additional file 1:** **Table S1.** Summary of seawater chemistry parameters represented as means ± s.e.during the experiment. Daily measurements of pH, temperature, salinity and total alkalinity were recorded within each tank. Levels of pCO_2_, Ω_Ca_ and Ω_Ar_ were calculated by the CO_2_SYS program based on measured levels of TA, pH, temperature, and salinity using the GEOSECS constants. **Table S2.** Summary statistics of alpha diversity from corals under different treatments. **Figure S1.** Principal coordinates analysis plot illustrates distinct bacterial assemblages in *G. fascicularis*, *A. valida* and seawater. Sequences that passed the quality filter were analyzed with PCoA based on unweighted UniFrac discrete distance. **Figure S2.** NMDS plots of Bray-Curtis distances between the microbial community composition:*G. fascicularis* and*A. valida*. Microbial function:*G. fascicularis* and*A. valida*. Each triangle represents different treatment groups. Area of polygons connecting samples from each group indicated the group stability: the smaller the area, the more stable. Correlation between community composition and function:*G. fascicularis* and*A. valida*. Changes in Bray–Curtis similaritiesof bacterial community composition using 16S rRNA genes and bacterial function using metatranscriptome profile. C: control; T: under stress; P: after stress; A: acidification; H: warming; AH: acidification-warming. **Figure S3**. Distribution of DEGs fold changes form* G. fascicularis*or* A. valida*. T: under stress; P: after stress; A: acidification; H: warming; AH: acidification-warming.

## Data Availability

The raw sequence information in this paper has been deposited in the NCBI SRA database under BioProject PRJNA402085.

## Abbreviations

A: Acidification
H: Warming
AH: Acidification and warming
DEGs: Differentially expressed genes
IPCC: Intergovernmental panel on climate change
SRA: Sequence read archive
OTUs: Operational taxonomic units
MG-RAST: Metagenomics rapid annotation using subsystem technology
FDR: False discovery rate
PD: Phylogenetic diversity
HSD: Honest significant difference
nMDS: Nonmetric multidimensional scaling
ANOSIM: Analysis of similarities
SIMPER: Similarity percentages
PERMDISP: Permutation of dispersion
PERMANOVA: Permutational multivariate analysis of variance
SEM: Standard errors of the means
IAA: Indole-3-acetic acid
IPA: Iso-propyl alcohol

## References

[1] Yuan X, Guo Y, Cai W-J, Huang H, Zhou W, Liu S (2019). Coral responses to ocean warming and acidifification: implications for future distribution of coral reefs in the South China Sea. Mar Pollut Bull.

[2] Pachauri RK, Allen MR, Barros VR, Broome J, Cramer W, Christ R et al. IPCC (2014) Climate change 2014: Synthesis report. In: Pachauri RK, Meyer LA, editors Contribution of working groups I, II and III to the fifth assessment report of the intergovernmental panel on climate change, Geneva, 2014. p. 151.

[3] Hoegh-Guldberg O, Mumby PJ, Hooten AJ, Steneck RS, Greenfield P, Gomez E (2007). Coral reefs under rapid climate change and ocean acidification. Science.

[4] Carpenter KE, Abrar M, Aeby G, Aronson RB, Banks S, Bruckner A (2008). One-third of reef-building corals face elevated extinction risk from climate change and local impacts. Science.

[5] Davy SK, Allemand D, Weis VM (2012). Cell biology of cnidarian-dinoflagellate symbiosis. Microbiol Mol Biol Rev.

[6] Brown BE, Bythell JC (2005). Perspectives on mucus secretion in reef corals. Mar Ecol Prog Ser.

[7] Rädecker N, Pogoreutz C, Voolstra CR, Wiedenmann J, Wild C (2015). Nitrogen cycling in corals: The key to understanding holobiont functioning?. Trends Microbiol.

[8] Van Alstyne KL, Schupp P, Slattery M (2006). The distribution of dimethylsulfoniopropionate in tropical Pacific coral reef invertebrates. Coral Reefs.

[9] Zhang F, Blasiak LC, Karolin JO, Powell RJ, Geddes CD, Hill RT (2015). Phosphorus sequestration in the form of polyphosphate by microbial symbionts in marine sponges. Proc Natl Acad Sci USA.

[10] Agostini S, Suzuki Y, Higuchi T, Casareto BE, Yoshinaga K, Nakano Y (2012). Biological and chemical characteristics of the coral gastric cavity. Coral Reefs.

[11] Ferrier-Pagès C, Houlbrèque F, Wyse E, Richard C, Allemand D, Boisson F (2005). Bioaccumulation of zinc in the scleractinian coral *Stylophora pistillata*. Coral Reefs.

[12] Rosenberg E, Koren O, Reshef L, Efrony R, Zilber-Rosenberg I (2007). The role of microorganisms in coral health, disease and evolution. Nat Rev Microbiol.

[13] Rosado PM, Leite DCA, Duarte GAS, Chaloub RM, Jospin G, Nunes da Rocha U (2019). Marine probiotics: increasing coral resistance to bleaching through microbiome manipulation. The ISME J.

[14] Glas B, Herndl GJ, Frade PR (2016). The microbiome of coral surface mucus has a key role in mediating holobiont health and survival upon disturbance. The ISME J.

[15] Reshef L, Koren O, Loya Y, Zilber-Rosenberg I, Rosenberg E (2006). The coral probiotic hypothesis. Environ Microbiol.

[16] Ahmed HI, Herrera M, Liew YJ, Aranda M (2019). Long-term temperature stress in the coral model Aiptasia supports the “anna karenina principle” for bacterial microbiomes. Front Microbiol.

[17] Li J, Bates KA, Hoang KL, Hector TE, Knowles SCL, King KC (2023). Experimental temperatures shape host microbiome diversity and composition. Glob Change Biol.

[18] Webster NS, Reusch TBH (2017). Microbial contributions to the persistence of coral reefs. The ISME J.

[19] Pootakham W, Mhuantong W, Putchim L, Yoocha T, Sonthirod C, Kongkachana W (2018). Dynamics of coral-associated microbiomes during a thermal bleaching event. Microbiol Open.

[20] McDevitt-Irwin JM, Baum JK, Garren M, Vega Thurber RL (2017). Responses of coral-associated bacterial communities to local and global stressors. Front Mar Sci.

[21] van Oppen MJH, Blackall LL (2019). Coral microbiome dynamics, functions and design in a changing world. Nat Rev Microbiol.

[22] Ziegler M, Seneca FO, Yum LK, Palumbi SR, Voolstra CR (2017). Bacterial community dynamics are linked to patterns of coral heat tolerance. Nat Commun.

[23] Zhou G, Cai L, Yuan T, Tian R, Tong H, Zhang W (2017). Microbiome dynamics in early life stages of the scleractinian coral *Acropora gemmifera* in response to elevated pCO_2_. Environ Microbiol.

[24] Pitts KA, Campbell JE, Figueiredo J, Fogarty ND (2020). Ocean acidification partially mitigates the negative effects of warming on the recruitment of the coral, *Orbicella faveolate*. Coral Reefs.

[25] Prada F, Caroselli E, Mengoli S, Brizi L, Fantazzini P, Capaccioni B (2017). Ocean warming and acidification synergistically increase coral mortality. Sci Rep.

[26] Agostini S, Fujimura H, Higuchi T, Yuyama I, Casareto BE, Suzuki Y (2013). The effects of thermal and high-CO_2_ stresses on the metabolism and surrounding microenvironment of the coral *Galaxea fascicularis*. CR Biol.

[27] Qin Z, Yu K, Liang Y, Chen B, Huang X (2020). Latitudinal variation in reef coral tissue thickness in the South China Sea: potential linkage with coral tolerance to environmental stress. Sci Total Environ.

[28] Warner ME, Fitt WK, Schmidt GW (1999). Damage to photosystem II in symbiotic dinoflagellates: a determinant of coral bleaching. Proc Natl Acad Sci USA.

[29] Bolger AM, Lohse M, Usadel B (2014). Trimmomatic: a flexible trimmer for Illumina sequence data. Bioinformatics.

[30] Schloss PD, Westcott SL, Ryabin T, Hall JR, Hartmann M, Hollister EB (2009). Introducing mothur: open-source, platform-independent, community-supported software for describing and comparing microbial communities. Appl Environ Microbiol.

[31] Caporaso JG, Kuczynski J, Stombaugh J, Bittinger K, Bushman FD, Costello EK (2010). QIIME allows analysis of high-throughput community sequencing data. Nat Methods.

[32] Meyer F, Paarmann D, D’Souza M, Olson R, Glass EM, Kubal M (2008). The metagenomics RAST server–a public resource for the automatic phylogenetic and functional analysis of metagenomes. BMC Bioinform.

[33] Wilke A, Bischof J, Harrison T, Brettin T, D’Souza M, Gerlach W (2015). A RESTful API for accessing microbial community data for MG-RAST. PLoS Comput Biol.

[34] Love MI, Huber W, Anders S (2014). Moderated estimation of fold change and dispersion for RNA-seq data with DESeq2. Genome Biol.

[35] Faith DP, Baker AM (2006). Phylogenetic diversity (PD) and biodiversity conservation: some bioinformatics challenges. Evolutionary Bioinform.

[36] Oksanen J, Kindt R, Legendre P, O’Hara B, Stevens MHH, Oksanen MJ (2007). The vegan package. Community Ecol Package.

[37] Sasabe J, Miyoshi Y, Rakoff-Nahoum S, Zhang T, Mita M, Davis BM (2016). Interplay between microbial d-amino acids and host d-amino acid oxidase modifies murine mucosal defence and gut microbiota. Nat Microbiol.

[38] Lin Z, Zheng X, Chen J (2023). Deciphering pHdependent microbial taxa and functional gene cooccurrence in the coral *Galaxea fascicularis*. Microb Ecol.

[39] Webster NS, Negri AP, Botté ES, Laffy PW, Flores F, Noonan S (2016). Host-associated coral reef microbes respond to the cumulative pressures of ocean warming and ocean acidification. Sci Rep.

[40] Zhu W, Wang H, Li X, Liu X, Zhu M, Wang A (2023). Consistent responses of coral microbiome to acute and chronic heat stress exposures. Mar Environ Res.

[41] Meron D, Atias E, Iasur Kruh L, Elifantz H, Minz D, Fine M (2011). The impact of reduced pH on the microbial community of the coral Acropora eurystoma. The ISME J.

[42] Vezzulli L, Brettar I, Pezzati E, Reid PC, Colwell RR, Hofle MG (2012). Long-term effects of ocean warming on the prokaryotic community: evidence from the vibrios. The ISME J.

[43] Yu X, Yu K, Liao Z, Liang J, Deng C, Huang W (2020). Potential molecular traits underlying environmental tolerance of *Pavona decussata* and *Acropora pruinosa* in Weizhou Island, northern South China Sea. Mar Pollut Bull.

[44] Liang J, Yu K, Wang Y, Huang X, Huang W, Qin Z (2017). Distinct bacterial communities associated with massive and branching scleractinian corals and potential linkages to coral susceptibility to thermal or cold stress. Front Microbiol.

[45] Putnam HM, Barott KL, Ainsworth TD, Gates RD (2017). The vulnerability and resilience of reef-building corals. Curr Biol.

[46] Simister RL, Deines P, Botté ES, Webster NS, Taylor MW (2012). Sponge-specific clusters revisited: a comprehensive phylogeny of sponge‐associated microorganisms. Environ Microbiol.

[47] Erwin PM, Pineda MC, Webster N, Turon X, Lopez-Legentil S (2014). Down under the tunic: bacterial biodiversity hotspots and widespread ammonia-oxidizing archaea in coral reef ascidians. The ISME J.

[48] Kublanov IV, Sigalova OM, Gavrilov SN, Lebedinsky AV, Rinke C, Kovaleva O (2017). Genomic analysis of *Caldithrix abyssi*, the thermophilic anaerobic bacterium of the novel bacterial phylum Calditrichaeota. Front Microbiol.

[49] Grottoli AG, Dalcin Martins P, Wilkins MJ, Johnston MD, Warner ME, Cai WJ (2018). Coral physiology and microbiome dynamics under combined warming and ocean acidification. PLoS One.

[50] O’Brien PA, Smith HA, Fallon S, Fabricius K, Willis BL, Morrow KM (2018). Elevated CO_2_ has little influence on the bacterial communities associated with the pH-tolerant coral, massive *Porites* spp. Front Microbiol.

[51] Thurber RV, Willner-Hall D, Rodriguez-Mueller B, Desnues C, Edwards RA, Angly F (2009). Metagenomic analysis of stressed coral holobionts. Environ Microbiol.

[52] Rädecker N, Pogoreutz C, Gegner HM, Cárdenas A, Roth F, Bougoure J (2021). Heat stress destabilizes symbiotic nutrient cycling in corals. Proc Natl Acad Sci USA.

[53] Spaepen S, Vanderleyden J. (2011). Auxin and plant-microbe interactions. In: Estelle M, Weijers D, Leyser O, Ljung K, editors. Cold Spring Harbor Perspectives in Biology, vol. 3. p. a001438. 10.1101/cshperspect.a001438 PMC3062209 21084388

[54] Maruyama A, Maeda M, Simidu U (1989). Microbial production of auxin indole-3-acetic acid in marine sediments. Mar Ecol Prog Ser.

