# Supplementary material for: The impacts of ocean acidification, warming and their interactive effects on coral prokaryotic symbionts
Source: Environ Microbiome. 2023 Jun 7;18:49. doi: 10.1186/s40793-023-00505-w (PMC10246149; doi:10.1186/s40793-023-00505-w)
Supplement: Supplementary file 1 — Additional file 1: Table S1. Summary of seawater chemistry parameters represented as means ± s.e.during the experiment. Daily measurements of pH, temperature, salinity and total alkalinity were recorded within each tank. Levels of pCO2, ΩCa and ΩAr were calculated by the CO2SYS program based on measured levels of TA, pH, temperature, and salinity using the GEOSECS constants. Table S2. Summary statistics of alpha diversity from corals under different treatments. Figure S1. Principal coordinates analysis plot illustrates distinct bacterial assemblages in G. fascicularis, A. valida and seawater. Sequences that passed the quality filter were analyzed with PCoA based on unweighted UniFrac discrete distance. Figure S2. NMDS plots of Bray-Curtis distances between the microbial community composition:G. fascicularis andA. valida. Microbial function:G. fascicularis andA. valida. Each triangle represents different treatment groups. Area of polygons connecting samples from each group indicated the group stability: the smaller the area, the more stable. Correlation between community composition and function:G. fascicularis andA. valida. Changes in Bray–Curtis similaritiesof bacterial community composition using 16S rRNA genes and bacterial function using metatranscriptome profile. C: control; T: under stress; P: after stress; A: acidification; H: warming; AH: acidification-warming. Figure S3. Distribution of DEGs fold changes form G. fascicularisor A. valida. T: under stress; P: after stress; A: acidification; H: warming; AH: acidification-warming. [file 40793_2023_505_MOESM1_ESM.docx]

**Supplemental material**:

**The impacts of ocean acidification, warming and their interactive effects on coral prokaryotic symbionts**

Jinlong Li, Guangjun Chai, Yilin Xiao, Zhiyong Li*

State Key Laboratory of Microbial Metabolism, School of Life Science and Biotechnology, Shanghai Jiao Tong University, Shanghai 200240, P. R. China

***Correspondence:**

Zhiyong Li: zyli@sjtu.edu.cn

**Table S1.** Summary of seawater chemistry parameters represented as means ± s.e. (min-max) during the experiment. Daily measurements of pH, temperature, salinity and total alkalinity (TA, measured by Orion™ Total Alkalinity Test Kit ThermoFisher) were recorded within each tank. Levels of pCO_2_, Ω_Ca_ and Ω_Ar_ were calculated by the CO_2_SYS program based on measured levels of TA, pH, temperature, and salinity using the GEOSECS constants.

| **Treatment** | **Salinity (‰)** | **Temperature(℃)** | **TA (μmol/kg SW)** | **pH(NTS)** | **pCO2 (microatm)** | **Ω**_ca_ | **Ω**_Ar_ |
| --- | --- | --- | --- | --- | --- | --- | --- |
| Control | 32 | 26.1±1.2 ( 23.0-26.7) | 2450 | 8.14±0.05(8.07-8.20) | 327±39(270-396) | 6.61±0.50(5.81-7.49) | 4.35±0.33(3.81-4.94) |
| Warming | 32 | 31.7±0.4(31.1-32.5) | 2450 | 8.07±0.04 (8.01-8.14) | 392±41(316-462) | 6.88±0.41 (6.37-7.75) | 4.61±0.28(4.27-5.19) |
| Acidifaction | 32 | 25.5±1.6(22.3-27.4) | 2450 | 7.68±0.04 (7.63-7.75) | 1133±100(943-1268) | 2.72±0.34 (2.19-3.33) | 1.79±0.23(1.42-2.20) |
| Warming & Acidification | 32 | 31.9±0.4 (31.0-32.4) | 2450 | 7.70±0.04 (7.64-7.74) | 1074±108(971-1262) | 3.53±0.27 (3.16-3.80) | 2.36±0.18 (2.12-2.55) |

**Table S2**. Summary statistics of alpha diversity from corals under different treatments

| ***G. fascicularis*** | **T** | | | | **P** | | | |
| --- | --- | --- | --- | --- | --- | --- | --- | --- |
|  | **C** | **A** | **H** | **AH** | **C** | **A** | **H** | **AH** |
| **Qualified seqs** | 63706±13563 | 58000±9226 | 36052±1221 | 64845±2150 | 48841±4677 | 84412±12966 | 80328±20448 | 99119±3772 |
| **Observed OTUs** | 942±102 | 1034±45 | 833±75 | 943±37 | 985±25 | 1020±15 | 818±59 | 865±112 |
| **chao1** | 1095±84 | 1123±52 | 971±76 | 1036±37 | 1088±17 | 1133±16 | 943±48 | 951±126 |
| **ACE** | 1048±88 | 1100±46 | 978±75 | 1023±32 | 1082±22 | 1116±11 | 932±47 | 933±122 |
| **Shannon** | 6.69±0.27 | 6.69±0.37 | 6.78±0.25 | 6.19±0.21 | 6.44±0.01 | 6.43±0.23 | 6.2±0.18 | 5.91±0.35 |
| **Simpson** | 0.95±0.01 | 0.95±0.02 | 0.97±0.01 | 0.93±0.01 | 0.93±0.01 | 0.94±0.01 | 0.95±0.01 | 0.93±0.02 |
| **PD whole tree** | 105.7±8.26 | 112.81±3.44 | 94.26±6.43 | 103.46±1.91 | 109.51±1.46 | 109.48±.08 | 95.65±4.32 | 95.29±10.06 |
| ***A. valida*** | **T** | | | | **P** | | | |
|  | **C** | **A** | **H** | **AH** | **C** | **A** | **H** | **AH** |
| **Qualified seqs** | 8593±217 | 10265±1637 | 17742±4766 | 10756±1935 | 20077±6632 | 12610±2849 | 12886±1353 | 12880±2868 |
| **Observed OTUs** | 169±9^ab^ | 138±4 | 125±11 | 242±28 | 143±10 | 391±29 | 302±77 | 146±20 |
| **chao1** | 202±3 | 154±9 | 151±17 | 302±41 | 207±24 | 488±28 | 393±108 | 182±20 |
| **ACE** | 205±9 | 155±8 | 159±18 | 309±40 | 195±18 | 489±36 | 384±102 | 189±28 |
| **Shannon** | 4.42±0.33 | 5.23±0.25 | 2.41±0.41* | 3.78±0.47 | 2.49±0.2 | 5.57±0.16** | 4.43±0.92* | 3.21±0.60 |
| **Simpson** | 0.89±0.02 | 0.95±0.01 | 0.61±0.08* | 0.78±0.07 | 0.68±0.03 | 0.94±0.01* | 0.82±0.09 | 0.68±0.08 |
| **PD whole tree** | 24.63±1.14 | 19.2±.8 | 20.25±1.03 | 33.73±3.4* | 23.1±1.1 | 50.67±1.92*** | 41.21±8.75** | 23.87±1.91 |

Note: OTUs, operational taxonomic units; PD whole tree, Phylogenetic Diversity whole tree. Average alpha diversity statistics ±s.e. calculated with alpha_diversity.py in QIIME. *(P<0.05), **(P<0.01), ***(P<0.001) indicate significant difference comparing with the control (TC or PC). C: control; T: under stress; P: after stress; A: acidification; H: warming; AH: acidification-warming.

**
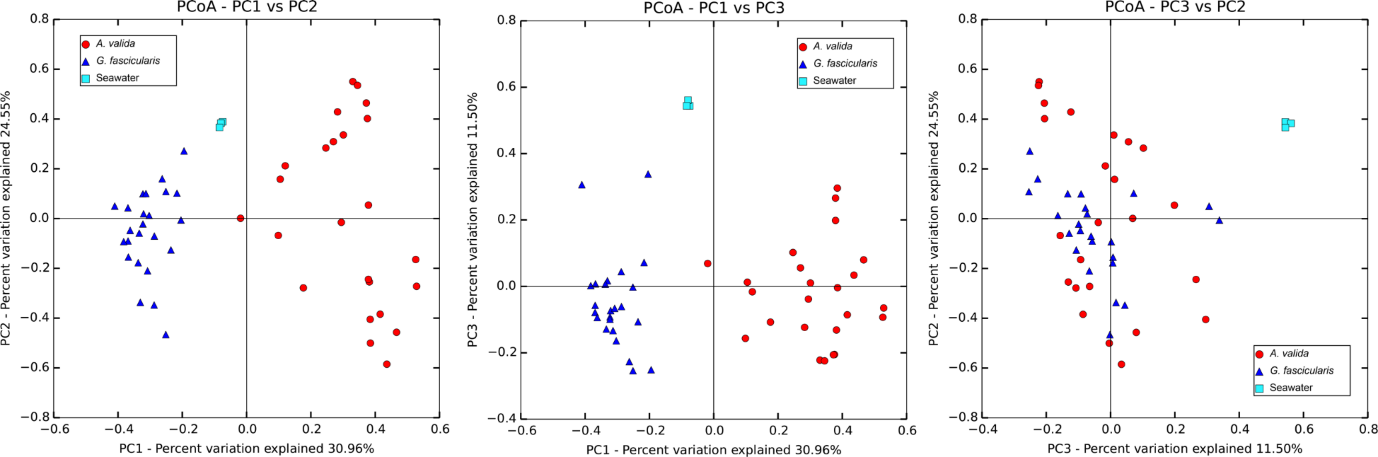
**

**Figure S1**. Principal coordinates analysis (PCoA) plot illustrates distinct bacterial assemblages in *G. fascicularis* (blue), *A. valida* (red) and seawater (cyan). Sequences that passed the quality ﬁlter were analyzed with PCoA based on unweighted UniFrac discrete distance.


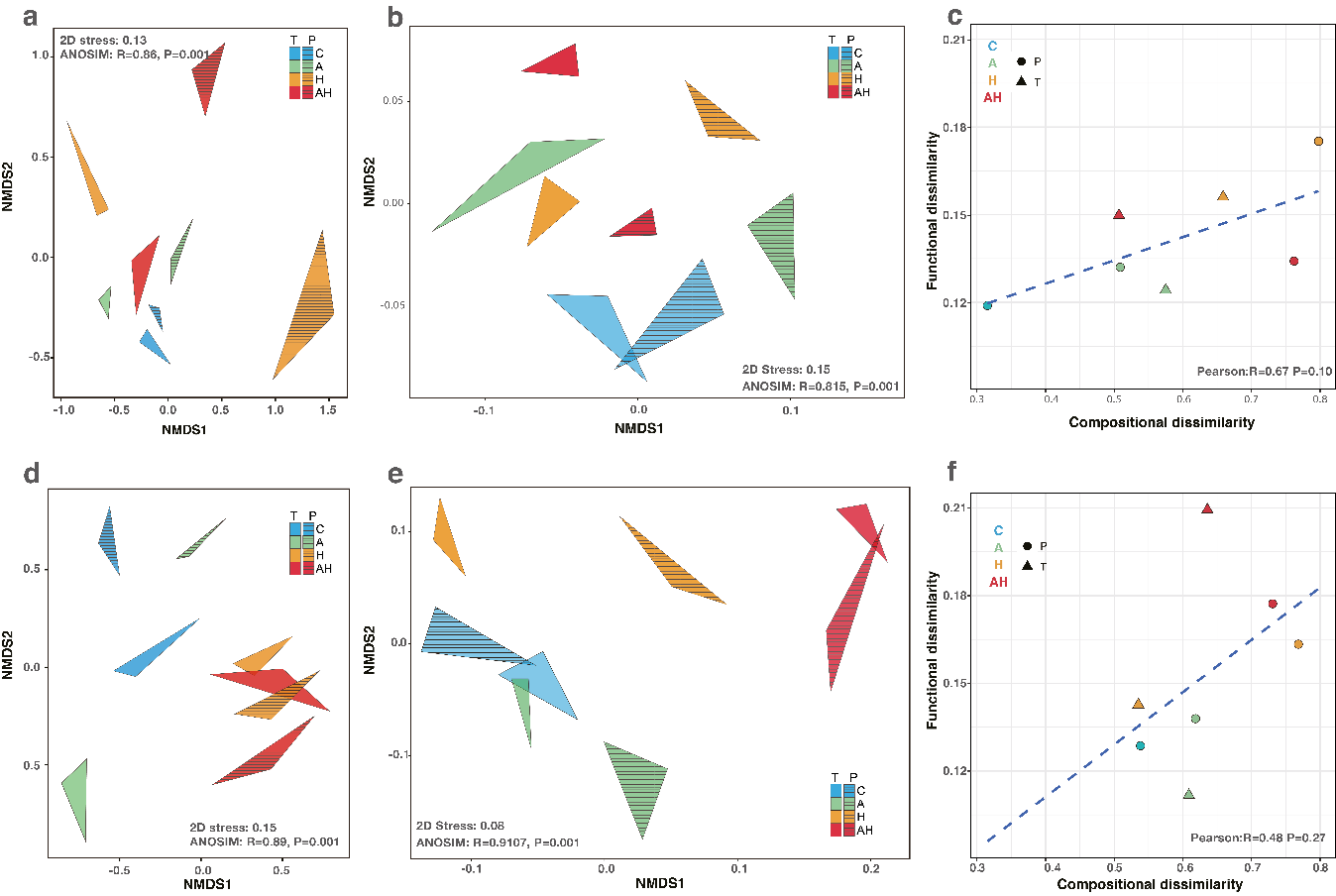


**Figure S2.** NMDS plots of Bray-Curtis distances between the microbial community composition: (a) *G. fascicularis* and (d) *A. valida*. Microbial function: (b) *G. fascicularis* and (e) *A. valida.* Each triangle represents different treatment groups. Area of polygons connecting samples from each group indicated the group stability: the smaller the area, the more stable. Correlation between community composition and function: (c) *G. fascicularis* and (f) *A. valida*. Changes in Bray–Curtis similarities (SIMPER) of bacterial community composition using 16S rRNA genes and bacterial function using metatranscriptome profile. C: control; T: under stress; P: after stress; A: acidification; H: warming; AH: acidification-warming.


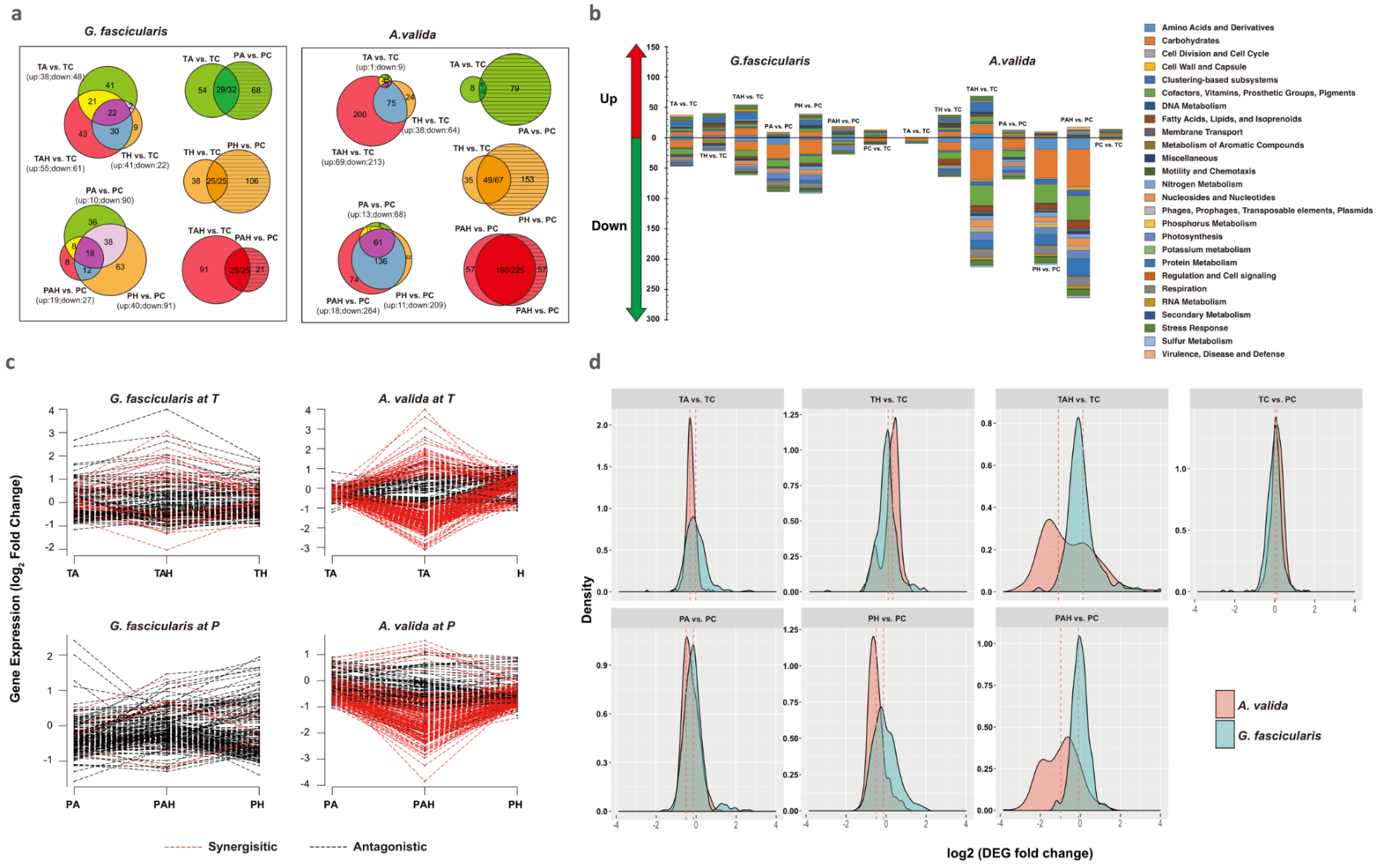


**Figure S3**. Distribution of DEGs fold changes form *G. fascicularis* (Blue) or *A. valida* (Red). T: under stress; P: after stress; A: acidification; H: warming; AH: acidification-warming.
